# Influence of injectate volume on paravertebral spread in erector spinae plane block: An endoscopic and anatomical evaluation

**DOI:** 10.1371/journal.pone.0224487

**Published:** 2019-10-28

**Authors:** You-Jin Choi, Hyun-Jin Kwon, Jehoon O, Tae-Hyeon Cho, Ji Yeon Won, Hun-Mu Yang, Shin Hyung Kim

**Affiliations:** 1 Division in Anatomy and Developmental Biology, Department of Oral Biology, Human Identification Research Institute, BK21 PLUS Project, Yonsei University College of Dentistry, Seoul, South Korea; 2 Department of Anatomy, Yonsei University College of Medicine, Seoul, South Korea; 3 Department of Anesthesiology and Pain Medicine, Anesthesia and Pain Research Institute, Yonsei University College of Medicine, Seoul, South Korea; Cleveland Clinic, UNITED STATES

## Abstract

The paravertebral spread that occurs after erector spinae plane block may be volume-dependent. This cadaveric study was undertaken to compare the extent of paravertebral spread with erector spinae plane block using different dye volumes. After randomization, twelve erector spinae plane blocks were performed bilaterally with either 10 ml or 30 ml of dye at the level of T5 in seven unembalmed cadavers except for two cases of unexpected pleural puncture using the 10 ml injection. Direct visualization of the paravertebral space by endoscopy was performed immediately after the injections. The back regions were also dissected, and dye spread and nerve involvement were investigated. A total of five 10 ml injections and seven 30 ml injections were completed for both endoscopic and anatomical evaluations. No paravertebral spread was observed by endoscopy after any of the 10-ml injections. Dye spread to spinal nerves at the intervertebral foramen was identified by endoscopy at adjacent levels of T5 (median: three levels) in all 30 ml injections. In contrast, the cases with two, four, and three out of five were stained at only the T4, T5, and T6 levels, respectively, with the 10 ml injection. Upon anatomical dissection, all blocks were consistently associated with posterior and lateral spread to back muscles and fascial layers, especially with the 30 ml injections, which showed greater dye expansion. In one 30 ml injection, sympathetic nerve involvement and epidural spread were observed at the level of the injection site. Although paravertebral spread following erector spinae plane block increased in a volume-dependent manner, this increase was variable and not pronounced. As the injectate volume increased for the erector spinae blocks, the injectate spread to the back muscles and fascial layers seemed to be predominantly increased compared with, the extent of paravertebral spread.

## Introduction

Conventional thoracic paravertebral block is a well-established technique for analgesia of the thoracic wall in various clinical settings, including thoracic surgery, breast surgery, rib fractures, and chronic neuropathic pain. However, there is a potential risk of pneumothorax or unintentional neuraxial injection[1–3]. Recently, an erector spinae plane (ESP) block with the, use of a more superficial needle placement than that used in the conventional method was introduced and is gaining popularity[4–8]. The ESP block targets the fascial plane deep to the erector spinae muscles at the tip of the transverse processes. Therefore, this technique is less likely to approach the pleura and incur attendant risks than the conventional method[4].

Although ESP blocks may provide simpler and safer regional anesthesia techniques than traditional paravertebral blocks[9], the optimal injectate volume required for achieving single or multilevel paravertebral spread is unknown. A 20 ml volume of injectate in unilateral ESP blocks seems to be typically used in clinical practice, despite a lack of published evidence regarding the optimal injectate volume[4–8]. Likewise, recent cadaveric studies evaluating injectate spread patterns following ESP blocks also used only 20 ml of injectate[10–12]. Although some conflicting results have been reported regarding paravertebral spread following EPS blocks[10], anatomical studies have consistently shown that a significant amount of injectate spreads to the back muscles or fascial layers with this technique[10–12]. These results imply that effective injectate spread to the paravertebral space following ESP block may be volume-dependent. To date, there has been no anatomical study assessing the difference in the extent of injectate spread following ESP block with different volumes of injectate, and as such, the influence of injectate volume on paravertebral spread with this technique has never been evaluated under controlled conditions.

We anticipated that a larger number of spinal nerves in the intervertebral foramen and deep paravertebral space would be stained after ESP blocks with 30 ml of injectate. Therefore, we designed this study to investigate paravertebral injectate spread in ESP blocks using 10 ml or 30 ml of dye in the mid-thoracic region of unembalmed cadavers. In this study, we sought to directly visualize the paravertebral space after ESP block using an endoscopic technique. Additionally, dye spread and nerve involvement according to the injectate volume were compared by anatomical dissection.

## Materials and methods

The Institutional Review Board approved the study for exemption from formal review (ref. No. 4-2018-0512). All cadavers used in the present study were legally donated to the Surgical Anatomy Education Centre at our institution. Twelve ESP blocks were conducted on the right and left sides of the posterior thoracic region in seven unembalmed cadavers except for two cases of unexpected pleural puncture using the 10 ml injection. Each cadaver underwent one ESP block with 10 ml of dye and one with 30 ml of dye, and the choice of which side was used for which volume was made randomly.

### Ultrasound-guided ESP block procedure

All cadavers were placed in a prone position, and injections were performed by a single, experienced anesthetist who specialized in regional anesthesia and pain medicine. A TE7 ultrasound unit (Mindray Bio-Medical Electronics, Shenzhen, China) with a high-frequency linear probe (4–16 MHz) and an 80-mm, 22-gauge needle was used for each cadaver. The needle was attached to an extension tube and connected to a syringe. The dye solution was either a 10.0 ml mixture of 9.0 ml distilled water, 0.7 ml latex solution, and 0.3 ml green ink, or a 30.0 ml mixture of 27.0 ml distilled water, 2.0 ml latex solution, and 1.0 ml green ink[12].

**Fig 1:**
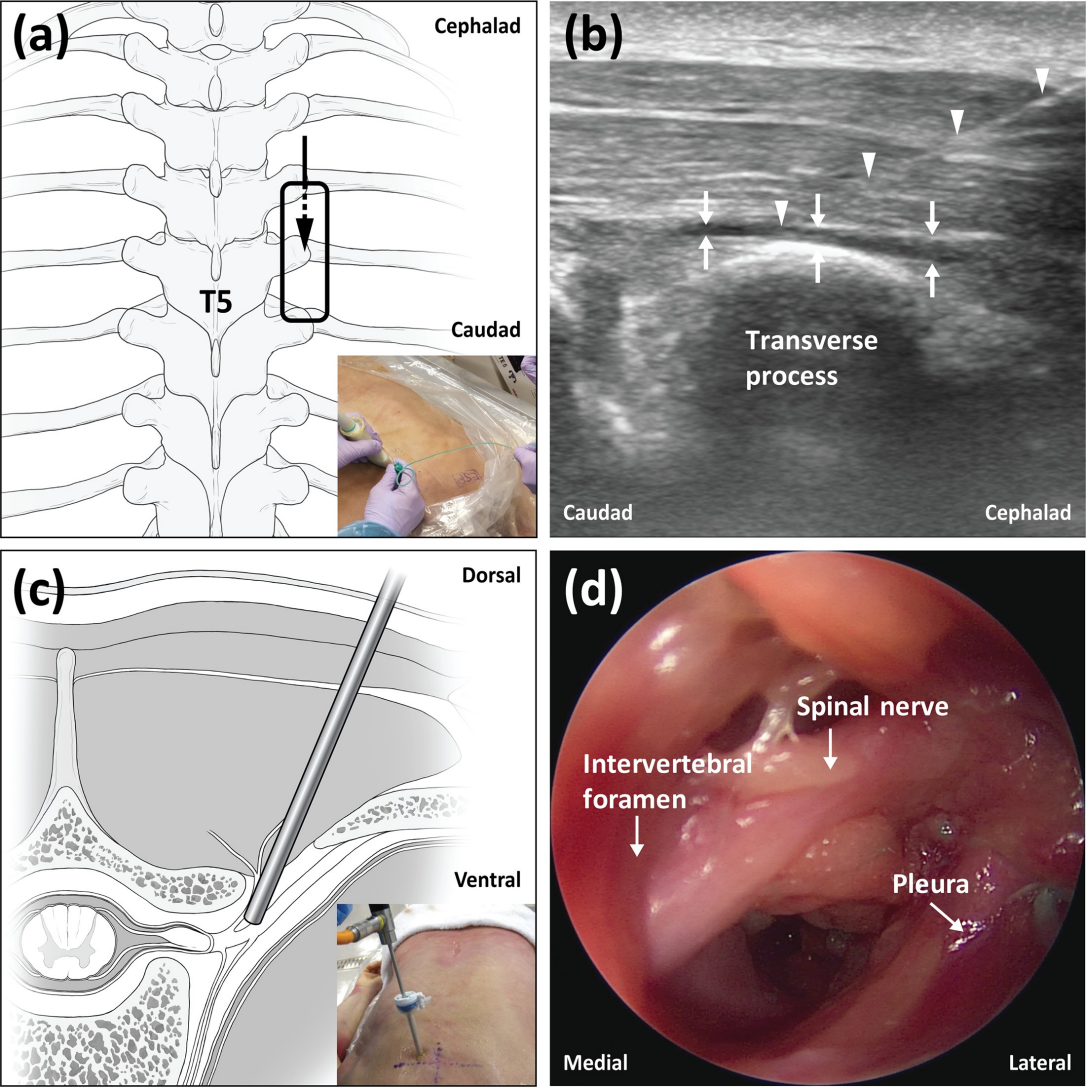
The experimental procedures for injection and endoscopy. (a) Schematic diagram showing the probe position and needle direction for the erector spinae plane block. (b) Ultrasound image demonstrating needle placement and dye spread of the erector spinae plane block. (c) Schematic diagram representing the endoscope position in the paravertebral space. (d) Typical endoscopic image of the thoracic paravertebral space.

Prescanning using both transverse and longitudinal views was performed sonographically to identify bony structures, including ribs, spinous processes, laminae, and transverse processes, and muscles, including the trapezius, rhomboid, and erector spinae muscles, at the level of the fifth thoracic vertebrae (T5). The probe was positioned longitudinally on the T5 transverse process, and anatomical structures were imaged in the sagittal plane. For the ESP block, the same in-plane technique with ultrasound guidance was used to advance the needle towards the tip of the T5 transverse process, in a cephalic to caudal direction, until contact was made with the transverse process (Fig 1A). The correct location of the needle tip in the fascial plane deep to the erector spinae muscles was confirmed by injecting 1.0 to 2.0 ml of normal saline and observing that the injected fluid lifted the erector spinae muscle off the transverse process without intramuscular injection (Fig 1B). After confirming the correct localization of the needle tip, 10 ml or 30 ml of dye solution, as described above, was injected. The injection speed for each 10 ml of injectate was controlled to a standardized period of 1 minute. After completion of the 10 ml or 30 ml injections, endoscopic evaluation of paravertebral spread was conducted at adjacent spinal levels.

### Endoscopic technique description

The technical method used for endoscopic evaluation of paravertebral spread in this study was based on a previously described technique[13] that used an endoscope system for arthroscopic surgery (LENS Surgical Imaging System, Smith & Nephew, London, UK). This technique was fully verified and practiced in a pilot test using an unembalmed cadaver before the current study was conducted. Ultrasound prescanning was performed to sonographically identify the midway point between neighboring transverse processes at adjacent levels of T5. These points were typically located 2.5–3.0 cm lateral from the midline. Then, expected entry points were marked on the skin of the cadavers at each spinal level. Before insertion of the trocar, its expected path was determined using a 25-gauge spinal needle that was inserted into the targeted paravertebral space. Using a sharp surgical blade, a small cut was carefully made for insertion of the trocar at the expected entry points. Then, a 6-mm diameter trocar with an introducer was advanced in a line perpendicular to the skin. The operator felt some resistance due to the costotransverse ligaments, and a tract between the skin and the paravertebral space was finally made after feeling a loss of resistance. After removal of the introducer, the trocar was angled slightly in the medial and cephalad direction, and then a rigid endoscope with a 4-mm diameter was inserted into the trocar (Fig 1C). Careful manipulation of the direction and depth of the endoscope allowed for visualization of the paravertebral space, including the major anatomical structures (Fig 1D). This technique did not cause the same anatomical disruptiveness observed with dissection; thus, images of the intact paravertebral space could be obtained, except at the trocar insertion site. Endoscopic views were easily obtained without the use of fluid or gas inflation for distending the space. The lack of a dense fascial covering and of fat tissue in the paravertebral space may have facilitated endoscopic examination. Additionally, in the cadaver model, the scarcity of vascularity and the presence of collapsed lungs induced by the prone position may have produced better visualization of the paravertebral space.

### Endoscopic and anatomical evaluation

The primary endpoint of this study was the number of stained spinal nerves in the intervertebral foramen within the paravertebral space for each volume of dye solution. During the insertion of the endoscope, the presence of a stained superior costotransverse ligament (SCTL) was first examined. Then, after passing the SCTL, we examined whether the T5 spinal nerve in the intervertebral foramen was stained. If the T5 nerve was stained, T4 and T6 nerve involvement at the intervertebral foramen was sequentially examined by endoscopy. Thus, endoscopic examination of the extent of dye spread was performed in the craniocaudal direction from the level of T5. During endoscopic evaluation, the extent of dye spread to anatomical structures within the paravertebral space was recorded by means of endoscopic imaging and videography. The number of stained thoracic spinal nerves at the intervertebral foramen for each volume of dye was expressed as the median (IQR [range]). After the completion of the endoscopic evaluation, anatomical dissection using a standard protocol was initiated immediately in all cadavers. After removing the subcutaneous layer, we exposed the external fasciae of the extrinsic back muscles. The extrinsic back muscles, such as the trapezius, latissimus dorsi, rhomboid, serratus posterior and splenius muscles were meticulously dissected. The intrinsic back muscles were also removed, preserving the costotransverse ligament, SCTL and external fasciae of the external intercostal muscle. The spreading patterns were recorded in this plane, and then posterior vertebral bodies were finally removed to identify the spreading patterns in the intercostal and paravertebral spaces. After or during the dissection, the anatomists recorded the spread patterns of the injected dye, and photographs were taken.

## Results

A total of seven unembalmed cadavers were included in this study. The subjects included three males and four females, with an average age of 79.6 years. All ESP blocks were successfully performed. Endoscopic procedures were sequentially performed to examine the paravertebral space. During trocar insertion or endoscope manipulation, two cases of unexpected pleural puncture using 10 ml of injectate led to leakage of body fluid into the paravertebral space that interfered with any further endoscopic procedure. Thus, these two cases were excluded from the analysis. Except for these two cases, typical endoscopic views of the paravertebral space containing thoracic spinal nerves at the intervertebral foramen were successfully obtained in all cadavers. After complete endoscopic evaluation, additional anatomical dissection was performed in all cases (10 ml, n = 5; 30 ml, n = 7). The results of the endoscopic and anatomical evaluations are summarized in Table 1.

**Table 1:** Comparison of dye staining at various spinal levels using 10 ml or 30 ml of injectate for erector spinae plane blocks.

	Volume of dye solution					
	10 ml injectate (n = 5)			30 ml injectate (n = 7)		
Spinal level	SCTL	Spinal nerves at the intervertebral foramen	Sympathetic nerves-epidural space	SCTL	Spinal nerves at the intervertebral foramen	Sympathetic nerves-epidural space
T2	-	-	-	1/7	-	-
T3	-	-	-	3/7	1/7	-
T4	2/5	-	-	6/7	4/7	-
T5 (injection site)	4/5	-	-	7/7	7/7	1/7
T6	3/5	-	-	7/7	5/7	-
T7	-	-	-	4/7	2/7	-
T8	-	-	-	1/7	-	-

‘-’ indicates no staining. SCTL is the superior costotransverse ligament. Values are the number of stained structures/total number of structures.

**Fig 2:**
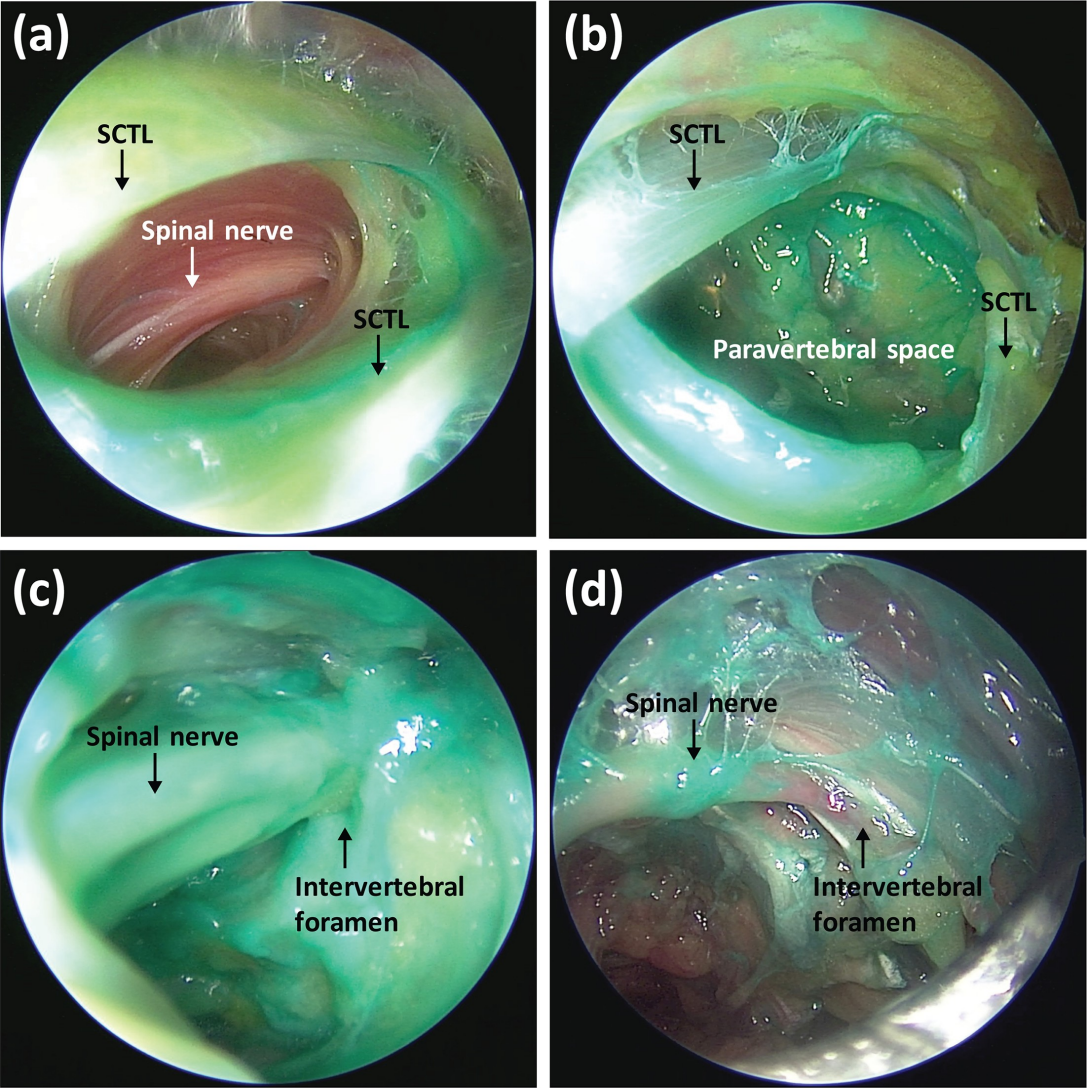
Endoscopic findings after erector spinae plane block. (a) A stained SCTL was identified at the level of T5, but no paravertebral spread was observed after injection with 10 ml of dye. (b) A stained SCTL at the level of T5 was identified, and paravertebral spread was also observed after injection with 30 ml of dye. (c) Deep staining of the spinal nerve in the intervertebral foramen at the T5 level was onserved after injection with 30 ml of dye. (d) A spinal nerve at the T7 level was stained after injection with 30 ml of dye, but the amount of dye was minute. SCTL is the superior costotransverse ligament.

For the endoscopic evaluation of ESP blocks using 10 ml of dye solution, stained SCTLs at the level of T4 to T6 were observed, but there was no paravertebral spread in any of the 10 ml blocks (Fig 2A). For the ESP blocks using 30 ml of dye solution, stained SCTLs at T2 to T8 and stained thoracic spinal nerves at the intervertebral foramina from T3 to T7 (primarily from T4 to T6) were observed by endoscopy (Fig 2B and 2C). The median number of stained thoracic spinal nerves at the intervertebral foramen was 3.0 (1.0–4.0 [1.0–6.0]) for 30-ml injections. The amount of dye at the intervertebral foramen observed by endoscopy gradually decreased distally from the T5 injection site (Fig 2D).

**Fig 3:**
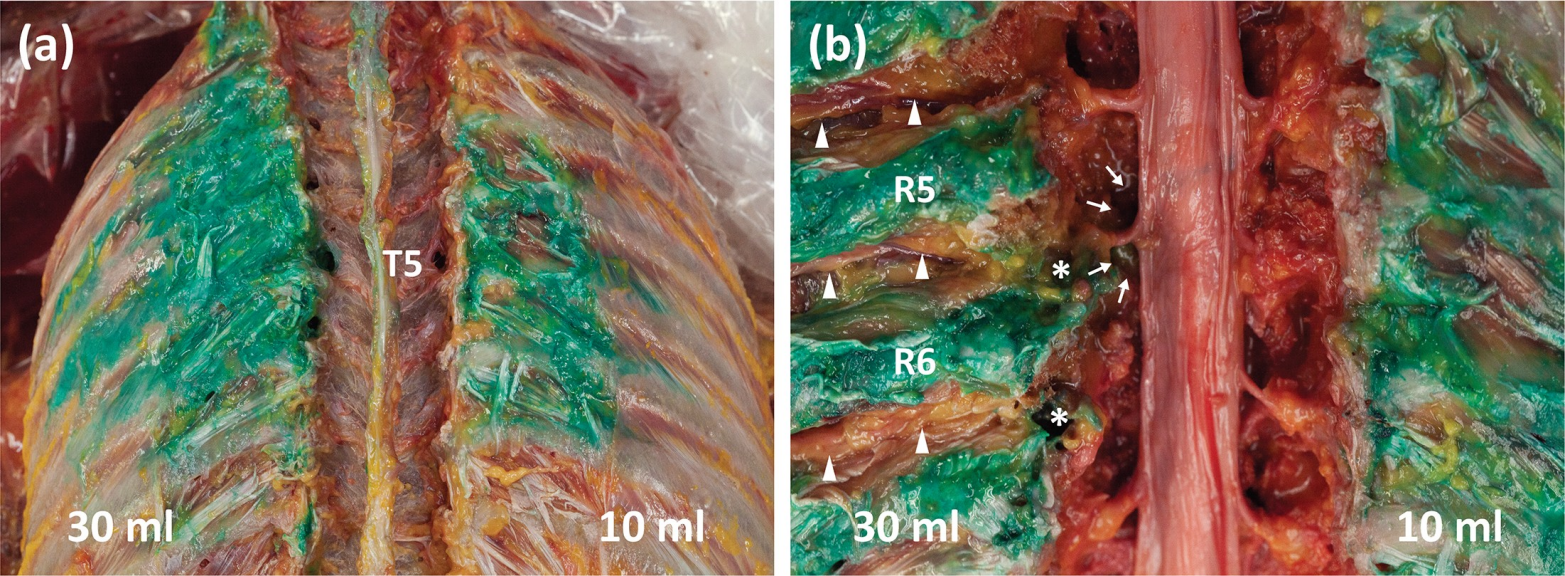
Anatomical dissection findings after erector spinae plane block. (a) Spread pattern of dye to the fascial layer of the external intercostal muscles after erector spinae plane block with 10 ml (right) and 30 ml (left) of dye. (b) Posterior vertebral bodies were removed. Using an injection of 10 ml (right) of dye, no paravertebral spread was observed. Using 30 ml (left) of dye, T5 and T6 spinal nerves in the intervertebral foraminal area were stained (asterisks), and epidural spread (arrows) was observed at the T5 level. Intercostal nerves were revealed laterally but were not stained (arrowheads).

For the anatomical dissection, most of the dye was located in the fascial layer of the erector spinae muscle group and external intercostal muscles in both ESP blocks (10 ml and 30 ml). In the 30 ml ESP blocks, we observed dye spread to posterior fascial layers of the erector spinae muscles in the craniocaudal direction, but dye spread was barely observed in the retrolaminar plane medially and vertically. Above all, lateral spread to the posterior layer of the thoracolumbar fascia and external intercostal muscles was predominantly observed when a larger extent of dye spread occurred using 30 ml of dye (Fig 3A). No dye penetrated the external intercostal muscles; therefore, no dye was observed in the space between the internal and innermost intercostal muscles, and no intercostal nerve involvement was observed regardless of the volume of dye used (Fig 3B). The number of stained thoracic spinal nerves in the intervertebral foremen was exactly the same with the endoscopic evaluation and the anatomical dissection. There was no clear intersegmental dye spread between adjacent vertebral levels within the paravertebral space in all blocks. In one 30 ml ESP block, sympathetic nerve involvement and epidural spread was observed, but they were limited to the T5 injection site level (Fig 3B).

## Discussion

In this cadaveric study, paravertebral spread was not observed in 10 ml ESP blocks. ESP blocks using 30 ml of injectate resulted in paravertebral spread to adjacent levels of the injection site; however, most of the injected dye spread to the posterior and lateral back muscles and the fascial layers.

A recent study of unilateral ESP blocks with 20 ml of injectate showed that the injectate spread to the intervertebral foramen over 2–3 spinal levels, which was confirmed with magnetic resonance imaging and anatomical dissection[11]. Similarly, our previous cadaveric study demonstrated that spinal nerves at the intervertebral foramen at 3.5 (median) spinal levels were stained in 20 ml ESP blocks[12]. However, contrary to our expectations, 30 ml ESP blocks did not result in more extensive paravertebral spread under endoscopic examination compared to the previous results obtained using 20 ml ESP blocks[12]. Previous cadaveric studies showed that paravertebral and intercostal spread with obvious and total somatic and sympathetic nerve involvement within these spaces could reach up to 3 to 4 vertebral levels from a single injection site in a conventional ultrasound-guided paravertebral block with 20 ml of injectate[14, 15]. Therefore, paravertebral spread following ESP block seems to be considerably limited compared to conventional paravertebral block. To summarize the current results, ESP blocks may volume-dependently lead to injectate spread to a more extensive area of the thoracic back region, but the extent of paravertebral spread following ESP blocks may not significantly increase by increasing the volume of injection beyond 20 ml at a single level.

The thoracic paravertebral space is a wedge-shaped area immediately adjacent to the thoracic vertebral column[13]. The paravertebral space seems not to be an anatomically isolated compartment or closed space[16, 17]. Injectate spread to the paravertebral space following ESP block may be possible through anticipated routes, such as SCTLs dorsally and/or intercostal spaces laterally[14]. In the present study, stained SCTLs at adjacent levels of the injection site were clearly identified by endoscopy in ESP blocks using both 10 ml and 30 ml of injectate. However, actual paravertebral spread via SCTLs only occurred when 30 ml of injectate was used, with no spread observed when 10 ml was used. This indicates that the majority of a 10-ml injection used for an ESP block ends up outside the paravertebral space and that this low volume used for an ESP block cannot lead to the penetration of dye to the paravertebral space via SCTLs. On the other hand, for all ESP blocks using 30 ml of injectate, intensively stained intrinsic back muscles, posterior layers of thoracolumbar fascia, and external intercostal muscles were all discernibly observed in a larger area. This finding indicates that the posterior rami of spinal nerves and lateral cutaneous branches of intercostal nerves may have been stained in this area[18, 19]. However, we could not find any clear intercostal nerve involvement in the intercostal space. Therefore, our results do not support the anatomical hypothesis that paravertebral spread may be possible through the intercostal space in ESP blocks. Additionally, the amount of dye within the paravertebral space in this study seemed to be too small to allow for lateral spread to the internal intercostal membrane. This finding is quite different from those associated with conventional paravertebral block, which results in lateral spread from the paravertebral to the intercostal space, with obvious intercostal nerve involvement at multiple vertebral segments[14, 15]. Even increasing the volume up to 30 ml for ESP blocks led to a predominate increase in posterior and lateral spread without deep intercostal spread, rather than an increase in medial spread to the SCTL, which could indicate a potential for paravertebral spread.

Interfascial plane blocks seem to be relatively safe from systemic toxicity that is associated with local anesthetics due to the low vascularity of fascial structures[20]. However, the potential for local anesthetic systemic toxicity was reported when performing ESP blocks with more than 40 ml of diluted local anesthetics [21]. However, safe dose ranges of local anesthetics for ESP blocks have not been tested using serum concentration measurements. Moreover, the results of our study indicate that continually increasing the volume of injectate for ESP blocks may not guarantee an increased extent of paravertebral spread. Therefore, for safe and effective ESP blocks, clinical data for optimal dose-volume regimens that consider patient conditions, injection sites, and types of local anesthetics, should be gathered for this technique.

There are several limitations in our current study. First, a small number of cadavers were used in this study. Additionally, postmortem changes in tissue integrity around the paravertebral space could affect the diffusion of the injectate. In living subjects, chest movement during inspiration and expiration can lead to delayed injectate diffusion to paravertebral or intercostal spaces. In cadavers, the insertion of an endoscopic device can result in tissue trauma of the costotransverse ligaments, thus potentially creating an artificial tract. This unintended consequence can make the dye flow into deeper layers. However, this study provided vivid *in situ* images of the paravertebral space, which could not be visualized by radiological evaluation or anatomical dissection, after ESP block.

In conclusion, ESP blocks with a low volume of injectate, such as 10 ml, do not result in paravertebral spread. Although paravertebral spread following ESP block volume-dependently increased in this study, injectate spread to the back muscles and fascial layers seemed to be more predominantly increased compared with the extent of paravertebral spread. These findings should be verified by evaluating the extent of sensory blocks and the actual analgesic effects following ESP blocks with different injectate volumes in living subjects.
